# Case Report: Acute Fulminant Cerebral Edema With Perivascular Abnormalities Related to Kawasaki Disease

**DOI:** 10.3389/fped.2021.732110

**Published:** 2021-09-17

**Authors:** Kenichi Maeda, Pin Fee Chong, Satoshi Akamine, Fumiya Yamashita, Yuya Morooka, Harushi Mori, Sooyoung Lee, Yumi Mizuno, Ryutaro Kira

**Affiliations:** ^1^Department of Pediatric Neurology, Fukuoka Children's Hospital, Fukuoka, Japan; ^2^Department of General Pediatrics and Interdisciplinary Medicine, Fukuoka Children's Hospital, Fukuoka, Japan; ^3^Department of Radiology, School of Medicine, Jichi Medical University, Tochigi, Japan; ^4^Department of Intensive Care, Fukuoka Children's Hospital, Fukuoka, Japan; ^5^Department of Pediatric Infectious Disease, Fukuoka Children's Hospital, Fukuoka, Japan

**Keywords:** acute encephalitis, perivascular lesions, acute encephalopathy, acute fulminant cerebral edema, acute brain swelling, vasogenic edema, Kawasaki disease, case report

## Abstract

**Introduction:** Kawasaki disease (KD) is an acute systemic vasculitis in children, but 0.4% of patients with KD exhibit central nervous system involvement. Acute encephalitis and encephalopathy accompanied with KD have been reported to be mostly self-limiting complications.

**Case Presentation:** A 2-year-old girl developed recurrent vomiting, a cluster of generalized seizures, and decreased consciousness on day 12 after the onset of KD. Magnetic resonance imaging (MRI) T2-weighted images on day 13 showed high signal intensities in bilaterally symmetrical and subcortical white matter and thalamus, and linear radial hyperintensities parallel to the cerebral vessels of the periventricular white matter. Diffuse white matter hyperintensity on the apparent diffusion coefficient map suggested vasogenic edema. Subsequently, lethal cerebral edema rapidly progressed in 8 hrs after the MRI examination.

**Conclusion:** To our knowledge, acute fulminant cerebral edema in patients with KD has not been previously reported. We should be aware of the possibility of severe encephalitis related to KD. Furthermore, diffuse white matter vasogenic edema with perivascular abnormalities on MRI may be an alerm, potentially leading to fatal cerebral edema.

## Introduction

Kawasaki disease (KD) is a systemic inflammatory disease that predominantly affects children younger than 5 years of age (1). The development of KD is caused by multiple factors: immunological responses, infectious triggers, environmental influences, and genetically predisposed hosts (2). Cardiovascular involvement is a major serious complication. Previous studies reported that 0.4% of KD cases exhibit central nervous system involvement, including aseptic meningitis, cerebral infarction, encephalitis, and acute encephalopathy (3). In most cases, encephalitis and encephalopathy are transient and self-limited, without sequelae (4, 5). We describe a child who developed severe encephalitis that rapidly progressed to lethal cerebral swelling related to KD. She demonstrated unique multiple periventricular linear radial lesions with diffuse vasogenic edema in the white matter on magnetic resonance imaging (MRI) prior to diffuse cerebral swelling.

## Case Report

A 2-year-old Japanese female with no history suggestive of any immune system or metabolic disorder visited our institute before the coronavirus disease (COVID-19) outbreak. She presented with fever 2 days before admission (day 1) and manifested cervical lymphadenopathy on day 2. On day 3, it was observed that she had a persistently high fever, numerous coalescent red spots on the trunk and limbs, peripheral edema, conjunctival injection, and a strawberry tongue. The initial laboratory evaluation showed leukocytosis (24,200/μL) and an elevated C-reactive protein (CRP) level (11.6 mg/dL). A diagnosis of KD was made, and she was admitted to our hospital where she was treated with intravenous immunoglobulin (IVIG), a dose of 2 g/kg/day, and oral aspirin. The recrudescent fever required another IVIG treatment (2 g/kg/day) on day 7. A high fever recurred on day 11, but no other clinical features of KD were present. Laboratory data revealed a decreased white blood cell (WBC) count (6,610 /μL) and CRP level (0.70 mg/dL).

On day 12, the patient experienced episodes of vomiting and a cluster of generalized tonic-clonic seizures without recovery of consciousness. The seizures resolved with multiple intravenous infusions of midazolam. The patient's body temperature was 40.1°C, and her blood pressure 107/58 mmHg. She did not have any upper respiratory tract symptoms or diarrhoea. Laboratory data showed a normal WBC count (7,620/μL), moderately increased CRP level (3.24 mg/dL), and mild hyponatremia (131 mEq/L). Serum glucose was 173 mg/dL, and alanine transaminase and aspartate aminotransferase levels were elevated at 110 and 185 IU/L, respectively. Cerebrospinal fluid (CSF) analysis did not show pleocytosis, but the protein level was elevated to 90 mg/dL. Blood and CSF culture were negative. The FilmArray® Meningitis and Encephalitis Panel (BioFire Diagnostics/Biomerieux, Salt Lake City, Utah), a pathogen-specific polymerase chain reaction testing of CSF capable of simultaneously detecting 6 bacteria, 7 viruses and a yeast, yielded negative results (6). Cranial computed tomography (CT) revealed no abnormalities. After convulsive status epilepticus, electroencephalography (EEG) showed a high-amplitude diffuse slowing background without ictal activity. During the interictal period, the patient could communicate with her parents and drink water.

**Figure 1:**
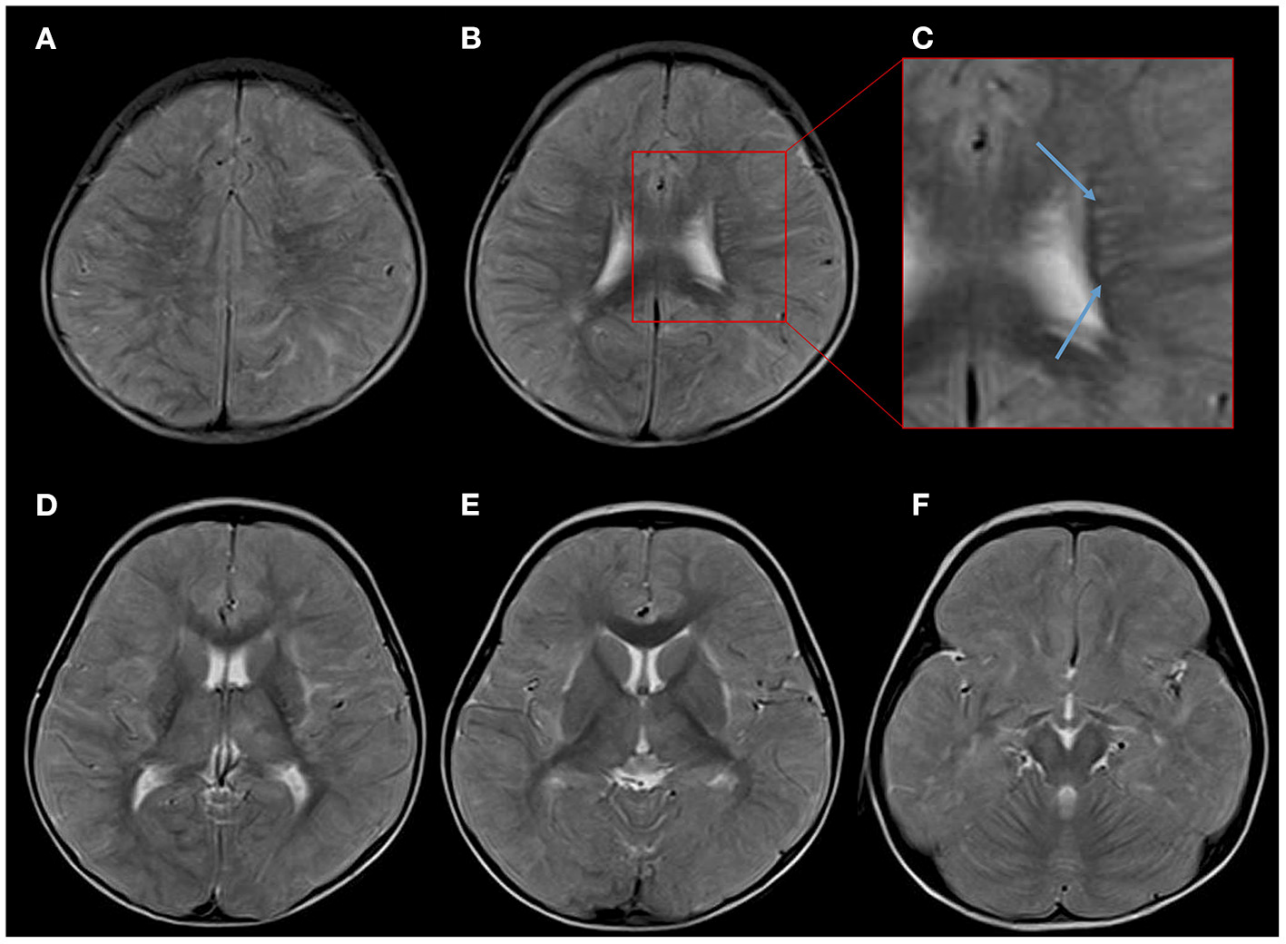
Overview of the axial T2-weighed images (T2WI) on day 13 after the onset of Kawasaki disease. **(A,B)** T2WI showing scattered high-intensity lesions mainly affecting the subcortical white matter. **(C)** An enlarged image showing the periventricular space revealing a radial pattern of hyperintensity along the deep cerebral veins (blue arrows). **(D–F)** White matter lesions observed bilaterally in the subcortical white matter, thalamus, and external capsule, and sparse in the internal capsule and corpus callosum.

**Figure 2:**
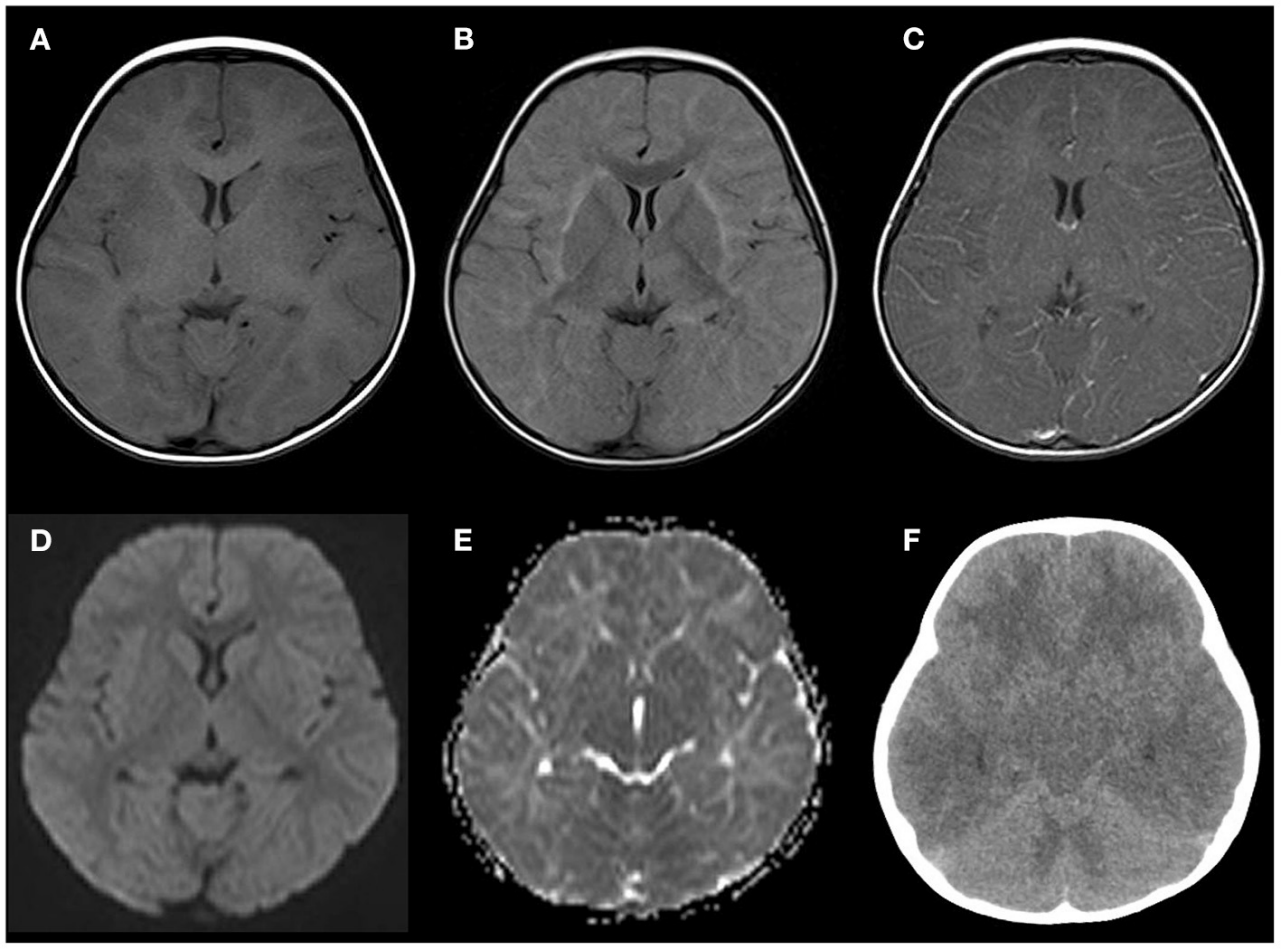
Neuroimaging findings on days 13–14. **(A–E)** Several sequences of the head MRI on day 13. **(A)** Isointense white matter lesions on a T1-weighted image (T1WI). **(B)** Fluid-attenuated inversion recovery image showing a high intensity in the subcortical white matter and external capsule. **(C)** Post-gadolinium T1WI showing no enhancement in the white matter or perivascular lesions. **(D)** Diffusion-weighted imaging showing isointense signals in the white matter lesions. **(E)** An apparent diffusion coefficient map showing diffuse hyperintensity in the white matter. **(F)** Brain computed tomography (CT) performed 8 h after the MRI demonstrating cerebral and cerebellar edema with sulcal and cisternal effacement.

Several convulsive seizures were observed on day 13. Moreover, the patient's mental status started to worsen and she became lethargic. MRI T2-weighted images (T2WI) showed bilateral symmetrical hyperintensities in the cerebral white matter and thalamus. The white matter lesions were observed predominantly in the subcortical white matter and sparsely in the internal capsule and corpus callosum (Figure 1). Linear radial hyperintensities parallel to the cerebral vessels of the periventricular space were also observed (Figure 1C). The white matter lesions were isointense on a T1-weighted image (T1WI), and there were hyperintense signals on fluid-attenuated inversion recovery (Figures 2A,B). The lesions showed no gadolinium enhancement (Figure 2C). The cerebral white matter displayed iso-intensity on diffusion-weighted images (Figure 2D), with high intensity on the apparent diffusion coefficient map (Figure 2E). These findings suggested vasogenic edema. Susceptibility-weighted images did not show any hemorrhages.

Eight hours after the MRI examination, the patient had abrupt cessation of spontaneous breathing that required intratracheal intubation and mechanical ventilation. Her pupillary light reflexes were absent, and her EEG showed diffuse low activity. Head CT demonstrated diffuse swelling of the cerebrum, cerebellum, and brainstem, with sulcal and cisternal effacement, suggesting brain herniation (Figure 2F). We diagnosed the patient with acute encephalitis related to KD based on the clinical manifestations, such as high fever, a cluster of seizures, vomiting, altered mental status, and neuroradiological abnormalities. We started intensive treatment with intravenous methylprednisolone, plasma exchange, mannitol, inotropic agents, and target temperature monitoring, but these were not effective for the brain swelling. The patient developed neurogenic pulmonary edema and central diabetes insipidus. The background activity on the EEG remained severely depressed, and the brainstem reflexes did not recover, and the patient died due to septic shock on day 41. Echocardiography was performed on days 3, 5, 6, 8, 11, and 13, and it did not show any coronary artery lesions. The additional laboratory tests for serum anti-myelin oligodendrocyte glycoprotein antibody and anti-N-methyl-D-aspartate receptor antibody in the CSF were negative.

## Discussion

Our case developed probable encephalitis, according to the International Encephalitis Consortium definition (7), during the subacute phase of KD. Neuroimaging revealed perivascular abnormalities and diffuse vasogenic edema in the cerebral white matter. The subsequent diffuse cerebral edema rapidly progressed, and the patient lost brainstem function within hours. Cerebral vascular lesions, such as stroke, aneurysms, or hemorrhage, were not observed. To the best of our knowledge, this is the first reported case of fatal encephalitis with severe cerebral edema related to KD.

Cases of life-threatening neurological conditions clinically adjudicated to be associated with KD are rare, and a few cases have been reported to involve cerebral vasculitis, aneurysms, encephalitis, or acute disseminated encephalomyelitis (ADEM) (8–13). There are limited reports of Kawasaki disease-related encephalitis. Three cases of clinically mild encephalitis/encephalopathy with a reversible splenial lesion which recovered completely were previously reported (4, 11, 12). Another case revealed transient subcortical white matter lesions showing favourable outcome (13). None of the cases showed a rapidly deteriorating course and fatal outcome such as the present case.

We postulate that two possible pathomechanisms might have been involved in the development of encephalitis in our patient. First, KD induces small-to-medium-sized vasculitis and endothelial dysfunction of microvessels, causing plasma leakage (9, 14). Vascular involvement with hyperpermeability may result in diffuse vasogenic edema. Another possible mechanism is inflammation or demyelination, which is shared with ADEM. The bilateral deep gray matter lesions and the symmetrical predominantly subcortical white matter lesions observed in this patient are common in patients with ADEM. In addition, perivenous patterns of demyelination and vasogenic edema as acute neuroinflammation are often observed in ADEM (15, 16). Therefore, these mechanisms may cause severe cerebral edema.

Acute fulminant cerebral edema (AFCE) has recently been recognized as a rare but devastating phenotype of suspected encephalitis (17). Four pediatric patients have been reported to have developed AFCE in association with COVID-19 or multisystem inflammatory syndrome (18). The case definition for AFCE was proposed as follows: fever, altered mental status, and/or new-onset seizures, followed by progression to diffuse cerebral edema as documented by neuroimaging and/or autopsy, with the exclusion of organic brain injury, metabolic disorders, or pre-existing neurological diseases (17). Our patient met the diagnostic criteria. Infectious triggers are speculated to be associated with AFCE, but the precise etiological mechanism is not fully understood. In our case, the core KD symptoms, except for a high fever, were not observed at the onset of the central nervous system symptoms. The para- or post-KD neurological events in our case suggest that the inflammatory process itself, not pathogens, causes AFCE.

MRI techniques have made it possible to distinguish between the two subtypes of brain edema: cytotoxic and vasogenic edema. Some reported AFCE cases show diffuse vasogenic edema of the whole brain, while others show cytotoxic edema of the cerebral cortex (18, 19). Another case showed diffuse T2WI hyperintensities at the same time of diffuse cerebral edema (20). Recently, Kawashima et al. reported a case of AFCE showing vasogenic edema of mainly the white matter with perivascular abnormalities before progressing to diffuse cerebral swelling (21). The MR images in our case were similar to those in their case. Although it is not yet known whether these different phenotypes on MRI imply heterogeneous mechanisms of developing cerebral edema or different stages of the same pathology, perivascular involvement might be a possible cue of secondary progression to fulminant cerebral edema.

In conclusion, we should be aware of the possibility of severe encephalitis related to KD, which may lead to fatal outcomes in patients. Diffuse white matter vasogenic edema with perivascular abnormalities on MRI may be an alarm, potentially leading to fatal cerebral edema, and it is necessary to consider the initiation of invasive interventions such as intracranial pressure monitoring.

## Data Availability Statement

The original contributions presented in the study are included in the article/supplementary material, further inquiries can be directed to the corresponding author/s.

## Ethics Statement

The studies involving human participants were reviewed and approved by ethics committee of the Fukuoka Children's Hospital. Written informed consent to participate in this study was provided by the participants' legal guardian/next of kin. Written informed consent was obtained from the minor(s)' legal guardian/next of kin for the publication of any potentially identifiable images or data included in this article.

## Author Contributions

KM, PC, and RK conceptualized and designed the study, drafted the initial manuscript, and reviewed and revised the manuscript. SA, FY, YMo, SL, and YMi performed the initial analyses and reviewed the revised manuscript. HM supervised the assessment of neuroimaging and critically reviewed the manuscript. All authors approved the final manuscript as submitted and agree to be accountable for all aspects of the work.

## Funding

This study was supported in part by a research grant from the Ministry of Health, Labour and Welfare of Japan (19HA1002 to RK) and the Japan Society for the Promotion of Science (grant number JSPS Kakenhi JP19K10613 to PC).

## Conflict of Interest

The authors declare that the research was conducted in the absence of any commercial or financial relationships that could be construed as a potential conflict of interest.

## Publisher's Note

All claims expressed in this article are solely those of the authors and do not necessarily represent those of their affiliated organizations, or those of the publisher, the editors and the reviewers. Any product that may be evaluated in this article, or claim that may be made by its manufacturer, is not guaranteed or endorsed by the publisher.
